# Supplementary material for: Association between the rs3812316 Single Nucleotide Variant of the MLXIPL Gene and Alpha-Linolenic Acid Intake with Triglycerides in Mexican Mestizo Women
Source: Nutrients. 2022 Nov 9;14(22):4726. doi: 10.3390/nu14224726 (PMC9692638; doi:10.3390/nu14224726)
Supplement: Supplementary file 1 [file nutrients-14-04726-s001.zip › Document S1. Informed consent.pdf]

INFORMED CONSENT

Guadalajara Jalisco, México. ________________,20__

According to the 4th constitutional article as published in the Official Journal of the Federation on January 3rd, 1983, the general law of health in the titles I, II, III, and IV, with all the corresponding chapters and articles, on the matter of the research for health and the NOM 004-SSA-2012.

We, Health Personnel of the Civil Hospital of Guadalajara "Fray Antonio Alcalde", General Service of Surgery and Molecular Biology on Medicine of the Doctorate in Sciences of Molecular Biology from the University of Guadalajara: Dr. Saúl Ocampo González, Dra. Bertha Ruíz Madrigal, and Q.F.B Lucia de la Cruz Color, seek your approval to participate in the research study:

"**CHARACTERIZATION OF THE DIFFERENTIAL EXPRESSION OF ChREBP-α AND ChREBP-β IN THE ADIPOSE AND HEPATIC TISSUES, AND ITS IMPLICATION ON LYPOGENESIS".**

I. In Mexico, over 70% of adults suffer from overweight or obesity, the complications associated with this illness include insulin resistance, fatty liver, high blood pressure, type 2 diabetes mellitus (T2DM), cardiovascular diseases, and cancer, among others. Thus, it is crucial to perform research that leads us to identify possible mechanisms to prevent these ailments and to ameliorate the state of health of all the affected people, in order to achieve an improvement in their quality of life.

Therefore, the aim of this study is to analyze the expression of the transcription factor ChREBP in hepatic as well as visceral and subcutaneous tissue, and its relationship with the development of obesity.

II. By participating in this study, you will be assigned to one of the groups according to your weight and height, you will be classified in either the normoweight or the obesity group. During your stay in the Civil Hospital Juan I. Menchaca, a sample of blood will be taken by venipuncture, as well as a small portion of your liver, visceral, and subcutaneous fat without provoking any disturbance or additional procedure to

the surgery. We will evaluate the expression of the aforementioned genes in these tissues.
We will perform several evaluations using several surveys to determine the relationship between genetic susceptibility, dietary habits, and lifestyle. Once the results are ready, you will be informed of our findings and you will receive any medical assistance you may need.
III. Among the benefits you will receive by participating in our study, you will perceive: nutritional evaluation and assistance based on your requirements by nutrition professionals completely free of charge. In addition, you will receive a report of the research results and a personal explanation to interpret them and resolve any questions you may have. However, there will not be any economic benefit from the researchers or the health professionals involved in the study toward the patients that enroll in the research.
IV. Within the possible complications associated with the study, you may experience the typical symptoms of the disease, such as mild abdominal pain or inflammation, which we would be able to quickly manage. In addition, the extraction of hepatic tissue may cause a feeling of malaise or pain in the wound site, mainly derived from gallbladder removal. However, this is generally considered a low-risk procedure.
V. If you were to feel uneasy due to some questions, doubts, comments, clarifications, or concerns, you are free and encouraged to speak to us at any moment. You are warranted to receive a full and clear answer to all the requested information.
VI. You are completely free to abandon the study at any time if you wish to do so. The clinical attention you receive will continue to be the same afterward.
VII. All the collected information will be kept in absolute confidence (we assure you that none of your personal data will be shared with third parties without your consent) and it will be handled exclusively by authorized health personnel related to the study. Likewise, all the data obtained will also be protected as strictly confidential.
VIII. We once again commit ourselves to providing you with the updated information obtained from this study. Also, we will let know if your liver and body fat samples were **not** extracted.

IX. If you were to present any inconvenience derived from the surgery, you may be legally entitled to receive the available medical treatment within the institution as compensation from the personnel in charge of the study.
X. If additional charges were to occur, these would be covered by the study budget.
All the questions related to the present study should be directed to Ph.D. Bertha Ruíz Madrigal or Ph.D. Montserrat Maldonado González through the phone number: 3331708699.
For all medical emergencies, please contact Dr. Saúl Ocampo González, Chief of Surgery and Clinical Nutrition, Jefe de Cirugía y Nutrición Clínica, ascribed to the General Service of Surgery through the phone number: 33 3942 4400.

NAME AND SIGNATURE OF PATIENT:

____

ADDRESS:

____

PLACE AND DATE:

____

NAME AND SIGNATURE OF THE RESPONSIBLE FOR RECEIVING AND ARCHIVING OF CONSENT:

____

WITNESS No. 1

NAME AND SIGNATURE

____

RELATIONSHIP TO PATIENT: ______________________________________

PLACE AND DATE:

____
