# Supplementary material for: Association between the rs3812316 Single Nucleotide Variant of the MLXIPL Gene and Alpha-Linolenic Acid Intake with Triglycerides in Mexican Mestizo Women
Source: Nutrients. 2022 Nov 9;14(22):4726. doi: 10.3390/nu14224726 (PMC9692638; doi:10.3390/nu14224726)
Supplement: Supplementary file 1 [file nutrients-14-04726-s001.zip › nutrients-1991874-supplementary.pdf]

Supplemental material

Table S1. Fatty acids intake in women and men

Variable	Total<br>N = 587	Women<br>N = 415	Men<br>N = 172	*p*-value
**SFA 4:0 (g)**	0.35 ± 0.38	0.33 ± 0.35	0.40 ± 0.44	0.075
**SFA 12:0 (g)**	0.44 ± 0.63	0.43 ± 0.69	0.48 ± 0.45	0.396
**SFA 17:0 (g)**	0.06 ± 0.45	0.06 ± 0.54	0.04 ± 0.06	0.530
**SFA 20:0 (g)**	0.05 ± 0.19	0.05 ± 0.22	0.05 ± 0.09	0.852
**SFA 22:0 (g)**	0.02 ± 0.06	0.03 ± 0.07	0.02 ± 0.06	0.476
**MUFA 20:1 (g)**	0.17 ± 0.82	0.18 ± 0.98	0.16 ± 0.15	0.882
**MUFA 22:1 (g)**	0.01 ± 0.04	0.01 ± 0.04	0.01 ± 0.04	0.724
**PUFA 18:4 (g)**	0.00 ± 0.08	0.01 ± 0.09	0.00 ± 0.02	0.714
**PUFA 20:5 (g)**	0.01 ± 0.04	0.01 ± 0.04	0.01 ± 0.03	0.762
**PUFA 22:5 (g)**	0.02 ± 0.04	0.02 ± 0.04	0.02 ± 0.04	0.872
**PUFA 22:6 (g)**	0.05 ± 0.13	0.05 ± 0.13	0.06 ± 0.13	0.565

Table S2. Baseline characteristics in the subgroup of subjects under cholecystectomy

Variable	Total<br>N = 24	Women<br>N = 18	Men<br>N = 6	*p*-value
**Age (y)**	35.1±10.6	35.2±7.9	35.0±17.0	0.975
**BMI (kg/m²)**	27.1±4.2	27.2±4.3	26.7±4.4	0.802
**Waist circumference (cm)**	86.0±11.2	83.1±10.1	93.3±11.4	0.774
**Body fat percentage (%)**	30.7±10.5	33.6±8.8	24.5±11.7	0.075
**Glucose (mg/dL)**	87.7±9.8	86.9±9.3	90.0±11.6	0.521
**Insulin (µU/mL)**	5.0±2.8	5.1±2.3	4.6±4.1	0.688
**HOMA-IR**	1.2±0.5	1.2±0.5	1.7±0.4	0.788
**Triglycerides (mg/dL)**	146.6±60.6	145.3±63.8	150.3±56.6	0.868
**Total cholesterol (mg/dL)**	159.6±52.7	169.2±47.9	132.5±60.7	0.147

**HDL-cholesterol (mg/dL)**	40.0±14.0	42.1±15.2	34.1±8.2	0.241
**LDL-cholesterol (mg/dL)**	93.4±47.2	102.3±43.9	68.1±51.0	0.131
**VLDL-cholesterol (mg/dL)**	29.2±12.2	28.8±12.8	30.1±11.6	0.831

Table S3: Anthropometric, biochemical, and nutritional variables according to the rs17145750 SNV´s of *MLXIPL* gene

	**Women**			**Men**		
**Variable**	**CC N = 342**	**CT+TT N = 73**	***p*-value**	**CC N = 148**	**CT+TT N = 24**	***p*-value**
**Age (y)**	42.06 ± 11.85	39.97 ± 11.17	0.170	39.30 ± 12.11	38.95 ± 12.4	0.898
**BMI (kg/m²)**	31.13 ± 7.06	31.88 ± 6.07	0.405	29.17 ± 6.18	30.15 ± 5.27	0.467
**Waist circumference (cm)**	94.52 ± 16.22	96.71 ± 15.05	0.298	97.70 ± 16.48	100.42 ± 16.33	0.454
**Hip circumference (cm)**	110.78 ± 14.64	111.89 ± 11.98	0.550	104.66 ± 11.12	108.15 ± 11.16	0.157
**WHR**	0.84 ± 0.08	0.85 ± 0.08	0.527	0.91 ± 0.08	0.90 ± 0.07	0.445
**Fat mass (g)**	33.04 ± 14.23	34.42 ± 12.71	0.455	25.19 ± 14.27	27.22 ± 13.17	0.513
**Body fat percentage (%)**	40.04 ± 9.02	40.98 ± 7.48	0.414	27.73 ± 9.59	27.84 ± 9.52	0.957
**Glucose (mg/dl)**	94.00 ± 17.38	91.01 ± 14.82	0.176	96.06 ± 22.42	93.08 ± 10.91	0.524
**Insulin**	13.41 ± 12.47	14.98 ± 15.07	0.360	10.92 ± 13.25	8.48 ± 5.23	0.406
**HOMA-IR**	3.14 ± 3.11	3.56 ± 4.45	0.347	2.55 ± 3.10	1.97 ± 1.35	0.399
**Triglycerides (mg/dl)**	140.87 ± 76.01	129.28 ± 63.18	0.305	148.11 ± 69.23	156.85 ± 80.30	0.678
**CT (mg/dl)**	187.74 ± 40.99	186.99 ± 48.07	0.891	186.92 ± 40.89	190.93 ± 33.08	0.649
**VLDL-c (mg/dl)**	28.17 ± 15.20	25.85 ± 12.63	0.228	29.62 ± 13.84	31.37 ± 16.05	0.575
**LDL-c (mg/dl)**	114.77 ± 38.18	118.35 ± 40.47	0.476	116.57 ± 36.85	115.76 ± 26.47	0.918
**HDL-c (mg/dl)**	44.79 ± 14.38	42.78 ± 12.47	0.271	40.72 ± 12.34	43.80 ± 13.02	0.263
**Non-HDL-c (mg/dl)**	142.94 ± 41.65	144.20 ± 45.36	0.819	146.19 ± 42.32	147.13 ± 32.60	0.918
**Energy (kcal)**	1855.65 ± 711.48	1866.65 ± 737.54	0.909	2397.57 ± 1004.74	2263.07 ± 926.64	0.540
**Carbohydrates (kcal)**	917.39 ± 372.59	933.96 ± 411.22	0.745	1172.74 ± 512.33	1156.36 ± 480.77	0.884
**Proteins (kcal)**	323.72 ± 126.59	310.45 ± 115.99	0.429	419.44 ± 173.16	384.99 ± 173.78	0.368

**Fat (kcal)**	613.22 ± 322.04	622.23 ± 291.14	0.833	805.38 ± 433.10	721.72 ± 381.58	0.375
**Carbohydrates (%)**	49.79 ± 9.63	49.98 ± 8.05	0.881	49.07 ± 9.42	51.66 ± 9.63	0.214
**Proteins (%)**	17.91 ± 4.87	17.13 ± 4.21	0.228	18.11 ± 4.24	17.23 ± 3.80	0.341
**Fat (%)**	32.19 ± 9.04	32.87 ± 7.67	0.564	32.81 ± 8.70	31.10 ± 8.05	0.370
**Saturated fat (g)**	22.93 ± 13.25	22.36 ± 11.27	0.743	30.22 ± 17.83	26.54 ± 15.75	0.342
**Monounsaturated fat (g)**	20.58 ± 11.46	22.22 ± 10.45	0.280	27.46 ± 16.49	24.85 ± 12.99	0.462
**Polyunsaturated fat (g)**	11.01 ± 7.77	12.24 ± 8.20	0.243	13.01 ± 10.26	12.78 ± 7.84	0.917
**SFA 4:0 (g)**	0.34 ± 0.36	0.31 ± 0.32	0.614	0.41 ± 0.46	0.34 ± 0.29	0.522
**SFA 6:0 (g)**	0.25 ± 0.26	0.24 ± 0.24	0.843	0.31 ± 0.35	0.27 ± 0.20	0.611
**SFA 8:0 (g)**	0.20 ± 0.20	0.22 ± 0.27	0.482	0.26 ± 0.28	0.22 ± 0.16	0.370
**SFA 10:0 (g)**	0.35 ± 0.34	0.35 ± 0.32	0.962	0.44 ± 0.42	0.41 ± 0.31	0.736
**SFA 12:0 (g)**	0.41 ± 0.48	0.53 ± 1.33	0.201	0.48 ± 0.47	0.46 ± 0.34	0.888
**SFA 14:0 (g)**	1.74 ± 1.42	1.64 ± 1.17	0.594	2.20 ± 1.77	2.08 ± 1.47	0.759
**SFA 16:0 (g)**	10.77 ± 6.24	11.06 ± 5.37	0.724	14.29 ± 8.94	13.36 ± 7.54	0.628
**SFA 17:0 (g)**	0.07 ± 0.60	0.03 ± 0.05	0.636	0.04 ± 0.06	0.03 ± 0.04	0.443
**SFA 18:0 (g)**	4.68 ± 30	4.88 ± 2.60	0.618	6.25 ± 4.23	5.85 ± 3.07	0.663
**SFA 20:0 (g)**	0.06 ± 0.24	0.04 ± 0.05	0.664	0.05 ± 0.10	0.03 ± 0.04	0.320
**SFA 22:0 (g)**	0.03 ± 0.07	0.02 ± 0.04	0.505	0.02 ± 0.06	0.01 ± 0.01	0.207
**MUFA 14:1 (g)**	0.06 ± 0.08	0.05 ± 0.05	0.341	0.08 ± 0.09	0.07 ± 0.08	0.629
**MUFA 16:1 (g)**	1.04 ± 0.73	1.01 ± 0.56	0.699	1.39 ± 1.17	1.33 ± 0.86	0.788
**MUFA 18:1 (g)**	17.96 ± 10.33	19.29 ± 9.05	0.329	24.04 ± 15.22	22.47 ± 11.88	0.630
**MUFA 20:1 (g)**	0.19 ± 1.09	0.13 ± 0.09	0.655	0.17 ± 0.16	0.16 ± 0.10	0.886
**MUFA 22:1 (g)**	0.01 ± 0.04	0.00 ± 0.01	**0.003**	0.01 ± 0.04	0.02 ± 0.06	0.293
**PUFA 18:2 (g)**	9.13 ± 6.83	10.43 ± 7.52	0.167	10.85 ± 9.54	10.83 ± 7.44	0.992
**PUFA 18:3 (g)**	0.89 ± 0.99	0.91 ± 0.58	0.858	1.05 ± 0.76	0.95 ± 0.42	0.524
**PUFA 18:4 (g)**	0.01 ± 0.11	0.00 ± 0.00	0.484	0.00 ± 0.02	0.01 ± 0.03	0.212
**PUFA 20:4 (g)**	0.13 ± 0.12	0.12 ± 0.09	0.565	0.16 ± 0.14	0.13 ± 0.09	0.331
**PUFA 20:5 (g)**	0.01 ± 0.04	0.01 ± 0.03	0.511	0.01 ± 0.03	0.02 ± 0.04	0.319
**PUFA 22:5 (g)**	0.02 ± 0.04	0.01 ± 0.02	**0.035**	0.01 ± 0.03	0.03 ± 0.05	0.230
**PUFA 22:6 (g)**	0.06 ± 0.14	0.03 ± 0.07	0.074	0.05 ± 0.12	0.08 ± 0.17	0.467
**Total n-3 (g)**	1.00 ± 1.13	0.98 ± 0.61	0.892	1.15 ± 0.78	1.11 ± 0.56	0.804
**Total n-6 (g)**	9.26 ± 6.86	10.55 ± 7.56	0.171	11.01 ± 9.56	10.96 ± 7.45	0.980

**n-3:n-6 ratio**	10.97 ± 6.50	10.99 ± 5.37	0.983	11.22 ± 9.22	10.44 ± 5.18	0.686
**Diet cholesterol (mg)**	268.13 ± 187.21	267.98 ± 163.91	0.995	356.53 ± 272.69	278.13 ± 169.08	0.174
**Dietary fiber (g)**	24.09 ± 12.01	23.66 ± 12.71	0.792	29.18 ± 15.71	30.07 ± 10.41	0.790
**Soluble fiber (g)**	0.48 ± 0.82	0.41 ± 0.72	0.574	0.70 ± 1.00	0.62 ± 0.91	0.720
**Insoluble fiber (g)**	1.06 ± 1.85	0.87 ± 1.63	0.449	1.61 ± 2.28	1.37 ± 2.01	0.632
**Crude fiber (g)**	4.64 ± 14.26	3.56 ± 4.21	0.543	5.73 ± 15.15	3.59 ± 3.20	0.494
**Total sugar (g)**	72.55 ± 39.52	77.28 ± 41.89	0.381	83.94 ± 46.74	85.55 ± 39.24	0.874
**Added sugars (g)**	21.64 ± 21.33	21.83 ± 19.35	0.957	19.24 ± 15.79	22.11 ± 19.40	0.530
**Glucose (g)**	8.12 ± 7.05	7.73 ± 7.29	0.689	8.15 ± 7.39	9.12 ± 9.48	0.569
**Galactose (g)**	0.51 ± 4.11	0.05 ± 0.15	0.360	0.50 ± 3.59	0.01 ± 0.03	0.504
**Fructose (g)**	10.85 ± 9.81	9.74 ± 9.91	0.403	11.46 ± 11.33	12.03 ± 13.76	0.826
**Sucrose (g)**	19.33 ± 21.39	21.13 ± 20.01	0.529	18.40 ± 16.17	20.78 ± 19.58	0.519
**Lactose (g)**	9.18 ± 10.24	9.43 ± 11.09	0.861	14.79 ± 15.85	9.51 ± 9.69	0.116

Table S4: Anthropometric, biochemical, and nutritional variables according to the rs3812316 SNV´s of *MLXIPL* gene

Variable	Women			Men		
	CC N = 357	CG+GG N = 58	*p*-value	CC N = 151	CG+GG N = 21	*p*-value
**Age (y)**	41.94 ± 11.99	40.44 ± 9.84	0.367	39.00 ± 11.97	40.19 ± 12.78	0.674
**BMI (kg/m^2)**	31.27 ± 6.92	31.95 ± 6.74	0.495	29.26 ± 6.21	31.11 ± 6.93	0.207
**Waist circumference (cm)**	94.92 ± 16.17	96.78 ± 15.76	0.418	97.88 ± 16.60	103.44 ± 18.97	0.160
**Hip circumference (cm)**	111.06 ± 14.32	112.11 ± 13.49	0.606	105.02 ± 11.14	107.90 ± 11.93	0.273
**WHR**	0.84 ± 0.07	0.86 ± 0.08	0.292	0.91 ± 0.08	0.94 ± 0.08	0.184
**Fat mass (g)**	33.28 ± 13.88	34.61 ± 14.52	0.513	25.38 ± 14.33	29.62 ± 16.41	0.214
**Body fat percentage (%)**	40.17 ± 8.89	40.85 ± 7.71	0.590	27.82 ± 9.66	28.91 ± 9.91	0.630
**Glucose (mg/dl)**	93.52 ± 17.4	91.98 ± 12.79	0.521	95.57 ± 22.48	95.04 ± 11.85	0.916
**Insulin**	13.57 ± 12.95	12.97 ± 11.86	0.746	10.75 ± 13.17	10.92 ± 6.75	0.957
**HOMA-IR**	3.20 ± 3.42	2.97 ± 2.79	0.640	2.50 ± 3.08	2.65 ± 1.87	0.845
**Triglycerides (mg/dl)**	142.27 ± 75.38	114.54 ± 52.70	**0.007**	147.68 ± 69.24	163.44 ± 78.84	0.398
**CT (mg/dl)**	189.04 ± 41.96	178.03 ± 42.75	0.065	188.00 ± 40.14	189.92 ± 31.19	0.833
**VLDL-c (mg/dl)**	28.45 ± 15.07	22.90 ± 10.53	**0.001**	29.53 ± 13.84	32.69 ± 15.76	0.338
**LDL-c (mg/dl)**	115.95 ± 39.17	112.29 ± 35.13	0.504	117.51 ± 36.10	114.32 ± 24.85	0.696
**HDL-c (mg/dl)**	44.64 ± 14.29	42.83 ± 12.31	0.363	40.95 ± 12.4	42.91 ± 13.14	0.501
**Non-HDL-c (mg/dl)**	144.40 ± 42.70	135.20 ± 38.53	0.124	147.04 ± 41.62	147.01 ± 31.15	0.997
**Energy (kcal)**	1865.10 ± 723.26	1886.00 ± 680.96	0.840	2444.41 ± 1009.83	2167.18 ± 726.92	0.227
**Carbohydrates (kcal)**	916.63 ± 379.37	967.10 ± 376.12	0.356	1197.39 ± 519.00	1098.23 ± 386.77	0.401
**Proteins (kcal)**	326.29 ± 127.40	298.27 ± 103.84	0.119	426.02 ± 175.12	380.47 ± 144.21	0.257
**Fat (kcal)**	620.91 ± 324.22	620.61 ± 278.66	0.995	820.98 ± 429.34	688.48 ± 339.30	0.178
**Carbohydrates (%)**	49.45 ± 9.54	51.44 ± 7.65	0.139	49.00 ± 9.23	51.24 ± 9.92	0.305
**Proteins (%)**	17.97 ± 4.80	16.25 ± 4.10	**0.011**	18.02 ± 4.14	17.65 ± 4.39	0.707

**Fat (%)**	32.46 ± 8.93	32.30 ± 7.61	0.894	32.97 ± 8.46	31.10 ± 8.41	0.345
**Saturated fat (g)**	23.21 ± 13.29	21.89 ± 10.95	0.483	30.85 ± 17.73	25.33 ± 13.96	0.173
**Monounsaturated fat (g)**	20.93 ± 11.64	21.82 ± 10.05	0.589	27.79 ± 16.34	24.84 ± 12.24	0.427
**Polyunsaturated fat (g)**	11.13 ± 7.94	13.16 ± 7.93	0.076	13.28 ± 10.27	12.05 ± 7.45	0.597
**SFA 4:0 (g)**	0.34 ± 0.36	0.30 ± 0.28	0.451	0.42 ± 0.46	0.30 ± 0.26	0.096
**SFA 6:0 (g)**	0.24 ± 0.260	0.24 ± 0.22	0.815	0.32 ± 0.35	0.24 ± 0.17	0.106
**SFA 8:0 (g)**	0.20 ± 0.20	0.22 ± 0.28	0.385	0.27 ± 0.28	0.21 ± 0.14	0.123
**SFA 10:0 (g)**	0.36 ± 0.34	0.35 ± 0.31	0.934	0.46 ± 0.42	0.37 ± 0.27	0.243
**SFA 12:0 (g)**	0.41 ± 0.48	0.55 ± 1.44	0.460	0.49 ± 0.47	0.42 ± 0.29	0.483
**SFA 14:0 (g)**	1.75 ± 1.41	1.62 ± 1.16	0.522	2.26 ± 1.78	1.96 ± 1.27	0.346
**SFA 16:0 (g)**	10.92 ± 6.34	10.96 ± 5.28	0.957	14.54 ± 8.92	13.07 ± 6.70	0.469
**SFA 17:0 (g)**	0.07 ± 0.59	0.03 ± 0.04	0.676	0.04 ± 0.06	0.03 ± 0.04	0.380
**SFA 18:0 (g)**	4.75 ± 3.05	4.79 ± 2.46	0.940	6.36 ± 4.20	5.67 ± 2.74	0.470
**SFA 20:0 (g)**	0.05 ± 0.24	0.05 ± 0.05	0.863	0.05 ± 0.10	0.03 ± 0.05	0.402
**SFA 22:0 (g)**	0.03 ± 0.07	0.02 ± 0.04	0.832	0.02 ± 0.06	0.01 ± 0.01	0.309
**MUFA 14:1 (g)**	0.06 ± 0.08	0.05 ± 0.05	0.149	0.08 ± 0.09	0.08 ± 0.08	0.864
**MUFA 16:1 (g)**	1.06 ± 0.74	0.94 ± 0.56	0.269	1.41 ± 1.17	1.38 ± 0.83	0.934
**MUFA 18:1 (g)**	18.26 ± 10.5	19.09 ± 8.75	0.576	24.39 ± 15.11	22.33 ± 11.2	0.550
**MUFA 20:1 (g)**	0.18 ± 1.06	0.13 ± 0.08	0.683	0.17 ± 0.16	0.13 ± 0.07	0.285
**MUFA 22:1 (g)**	0.01 ± 0.04	0.00 ± 0.00	**< 0.001**	0.01 ± 0.04	0.01 ± 0.03	0.646
**PUFA 18:2 (g)**	9.23 ± 6.97	11.42 ± 7.61	**0.033**	11.11 ± 9.58	10.13 ± 7.18	0.652
**PUFA 18:3 (g)**	0.90 ± 0.98	0.92 ± 0.51	0.929	1.05 ± 0.75	0.94 ± 0.36	0.250
**PUFA 18:4 (g)**	0.01 ± 0.10	0 ± 0.01	0.567	0.00 ± 0.02	0.01 ± 0.02	0.651
**PUFA 20:4 (g)**	0.13 ± 0.12	0.11 ± 0.08	0.292	0.17 ± 0.13	0.1 ± 0.07	**0.003**
**PUFA 20:5 (g)**	0.01 ± 0.04	0.01 ± 0.03	0.508	0.01 ± 0.04	0.01 ± 0.02	0.515
**PUFA 22:5 (g)**	0.02 ± 0.04	0.01 ± 0.01	**0.002**	0.02 ± 0.04	0.01 ± 0.03	0.718
**PUFA 22:6 (g)**	0.06 ± 0.13	0.03 ± 0.05	**0.018**	0.06 ± 0.13	0.03 ± 0.10	0.355
**Total n-3 (g)**	1.02 ± 1.11	0.98 ± 0.54	0.815	1.17 ± 0.78	1.02 ± 0.39	0.380
**Total n-6 (g)**	9.37 ± 7.01	11.54 ± 7.64	**0.035**	11.28 ± 9.60	10.23 ± 7.19	0.631
**n-3:n-6 ratio**	10.84 ± 6.43	12.19 ± 5.95	0.144	10.93 ± 8.09	10.47 ± 5.46	0.804
**Diet cholesterol (mg)**	271.44 ± 187.16	254.41 ± 157.89	0.520	369.06 ± 270.48	250.74 ± 164.74	0.053
**Dietary fiber (g)**	24.06 ± 12.13	24.35 ± 12.11	0.869	29.60 ± 15.62	28.6 ± 9.710	0.776

**Soluble fiber (g)**	0.44 ± 0.79	0.52 ± 0.79	0.496	0.69 ± 1.03	0.61 ± 0.66	0.727
**Insoluble fiber (g)**	0.99 ± 1.78	1.12 ± 1.87	0.611	1.6 ± 2.33	1.37 ± 1.54	0.662
**Crude fiber (g)**	4.68 ± 13.94	3.02 ± 1.71	0.374	5.63 ± 15.06	3.83 ± 3.21	0.586
**Total sugar (g)**	72.70 ± 40.64	77.18 ± 37.11	0.440	84.48 ± 47.11	87.95 ± 35.44	0.747
**Added sugars (g)**	22.30 ± 21.91	19.19 ± 14.74	0.392	19.39 ± 15.73	19.73 ± 19.13	0.940
**Glucose (g)**	8.00 ± 7.03	8.12 ± 7.39	0.913	8.08 ± 7.66	8.47 ± 7.87	0.829
**Galactose (g)**	0.49 ± 3.98	0.05 ± 0.16	0.421	0.49 ± 3.56	0.01 ± 0.03	0.540
**Fructose (g)**	10.61 ± 9.71	10.20 ± 10.07	0.773	11.17 ± 11.36	11.7 ± 13.17	0.845
**Sucrose (g)**	19.68 ± 21.84	19.00 ± 16.58	0.825	18.49 ± 16.37	22.66 ± 18.90	0.287
**Lactose (g)**	9.17 ± 10.41	9.34 ± 10.74	0.916	14.54 ± 15.54	11.93 ± 13.24	0.465

Table S5: Frequencies of dyslipidemias according to the rs17145750 and rs3812316 SNV´s of *MLXIPL* gene in women

	rs17145750 C>T			rs3812316 C>G		
	**CC**	**CT+TT**	***p*-value**	**CC**	**CG+GG**	***p*-value**
**Hypercholesterolemia (%)**	34.7	31.9	0.684	36.4	24.1	0.075
**Elevated LDL-c (%)**	66.5	69.4	0.680	67.2	65.5	0.880
**Low HDL-C (%)**	39.5	45.8	0.355	40.6	41.4	1.000
**Hypertriglyceridemia (%)**	36.5	30.6	0.347	38.1	19.0	**0.005**

LDL-c: Low-density lipoprotein cholesterol, HDL-c: High-density lipoprotein cholesterol

Table S6: Frequencies of dyslipidemias according to the rs17145750 and rs3812316 SNV´s of *MLXIPL* gene in men

	rs17145750 C>T			rs3812316 C>G		
	**CC**	**CT+TT**	***p*-value**	**CC**	**CG+GG**	***p*-value**
**Hypercholesterolemia (%)**	34.2	29.2	0.653	33.8	28.6	0.806
**Elevated LDL-c (%)**	68.2	66.7	1.000	69.3	71.4	1.000
**Low HDL-C (%)**	51.0	37.5	0.273	49.7	47.6	1.000
**Hypertriglyceridemia (%)**	41.6	45.8	0.824	40.4	52.4	0.348

LDL-c: Low-density lipoprotein cholesterol, HDL-c: High-density lipoprotein cholesterol

Table S7. Logistic regression models of hypertriglyceridemia in women

		B	*p*-value	OR	95% CI	
					**Inferior limit**	**Superior limit**
Model 1	HDL-cholesterol (mg/dL)	-0.066	< 0.001	0.936	0.915	0.958
	Constant	2.472	< 0.001	11.842		
Model 2	Total cholesterol (mg/dL)	0.019	< 0.001	1.020	1.012	1.027
	HDL-cholesterol (mg/dL)	-0.083	< 0.001	0.921	0.898	0.945
	Constant	-0.581	0.439	0.559		
Model 3	Total cholesterol (mg/dL)	0.021	< 0.001	1.021	1.013	1.029
	HDL-cholesterol (mg/dL)	-0.072	< 0.001	0.930	0.906	0.955
	Waist circumference (cm)	0.040	< 0.001	1.041	1.022	1.060
	Constant	-5.204	< 0.001	0.005		
Model 4	Glucose (mg/dL)	0.030	0.026	1.030	1.004	1.057
	Total cholesterol (mg/dL)	0.021	< 0.001	1.022	1.014	1.030
	HDL-cholesterol (mg/dL)	-0.072	< 0.001	0.931	0.907	0.955
	Waist circumference (cm)	0.037	< 0.001	1.038	1.018	1.058
	Constant	-7.743	< 0.001	0.000		
Model 5	Glucose (mg/dL)	0.030	0.029	1.030	1.003	1.058
	Total cholesterol (mg/dL)	0.021	< 0.001	1.021	1.013	1.030
	HDL-cholesterol (mg/dL)	-0.072	< 0.001	0.931	0.907	0.955
	Waist circumference (cm)	0.039	< 0.001	1.039	1.020	1.060
	SNV´s rs3812316 (CC *vs* CG+GG)	-1.042	0.020	0.353	0.147	0.846
	Constant	-7.725	< 0.001	0.000		

Table S8. Logistic regression models of hypertriglyceridemia in men

		B	*p*-value	OR	95% CI	
					**Inferior limit**	**Superior limit**
Model 1	HDL-cholesterol (mg/dL)	-0.114	< 0.001	0.892	0.849	0.939
	Constant	4.171	< 0.001	64.754		
Model 2	Total cholesterol (mg/dL)	0.022	0.001	1.023	1.010	1.036
	HDL-cholesterol (mg/dL)	-0.127	< 0.001	0.881	0.833	0.932
	Constant	0.363	0.804	1.438		
Model 3	Total cholesterol (mg/dL)	0.024	0.001	1.024	1.010	1.038
	HDL-cholesterol (mg/dL)	-0.125	< 0.001	0.883	0.833	0.936
	Protein intake (g)	-0.016	0.010	0.984	0.972	0.996
	Constant	1.716	0.293	5.564		
Model 4	Total cholesterol (mg/dL)	0.021	0.003	1.021	1.007	1.035
	HDL-cholesterol (mg/dL)	-0.107	< 0.001	0.898	0.846	0.953
	Fat mass (kg)	0.061	0.031	1.063	1.005	1.124
	Protein intake (g)	-0.014	0.026	0.986	0.973	0.998
	Constant	-0.333	0.862	0.717		

Table S9. Multiple lineal regression of parameters associated with triglyceride levels in women

	B	Standard error	95% CI	*p*-value
***Model 1,* R^2 = 18.9**				
HDL-cholesterol	-0.006	0.001	-0.008 – -0.005	< 0.001
***Model 2,* R^2 = 32.2**				
HDL-cholesterol	-0.007	0.001	-0.008 – -0.006	< 0.001
Total cholesterol	0.002	0.000	0.001 – 0.002	< 0.001
***Model 3,* R^2 = 63.1**				
HDL-cholesterol	-0.006	0.001	-0.007 – -0.004	< 0.001
Total cholesterol	0.002	0.000	0.001 – 0.002	< 0.001
Waist circumference	0.004	0.001	0.002 – 0.005	< 0.001
***Model 4,* R^2 = 64.3**				
HDL-cholesterol	-0.006	0.001	-0.007 – -0.004	< 0.001
Total cholesterol	0.002	0.000	0.001 – 0.002	< 0.001
Waist circumference	0.004	0.001	0.003 – 0.005	< 0.001
SNV´s rs3812316 C/G	-0.073	0.028	-0.127 – -0.019	0.008

HDL-cholesterol: High-density lipoprotein cholesterol, CI: Confidence Interval.

Table S10. Multiple lineal regression of parameters associated with triglyceride levels in men

	B	Standard error	95% IC	*p*-value
Model 1, **R^2 = 58.3**				
HDL-cholesterol	-0.010	0.002	-0.014 – -0.007	< 0.001
Model 2, **R^2 = 70.4**				
HDL-cholesterol	-0.008	0.002	-0.011 – -0.005	< 0.001
Body fat percentage	0.008	0.002	0.005 – 0.012	< 0.001
Model 3, **R^2 = 74.0**				
HDL-cholesterol	-0.008	0.002	-0.011 – -0.005	< 0.001
Body fat percentage	0.008	0.002	0.005 – 0.012	< 0.001
Albumin	0.092	0.033	0.026 – 0.157	0.007
Model 4, **R^2 = 77.6**				
HDL-cholesterol	-0.007	0.001	-0.010 – -0.004	< 0.001
Body fat percentage	0.007	0.002	0.004 – 0.011	< 0.001
Albumin	0.118	0.032	0.054 – 0.182	< 0.001
Total dietary fiber	-0.004	0.001	-0.007 – -0.001	0.004

HDL-cholesterol: High-density lipoprotein cholesterol, CI: Confidence Interval.
